# Obésité de l’adolescent à Abidjan: prévalence et facteurs associés-résultats d’une enquête en milieu scolaire dans la commune de Koumassi

**DOI:** 10.11604/pamj.2025.52.37.46052

**Published:** 2025-09-24

**Authors:** Tiépé Rokia Ouattara, Hervé Micondo, Franck Kouassi, Félicia Akousssi, Adelaïde Hue-Lou, Jocelyne Danho, Anselme N'guessan, Assita Yao, Kevin Acho, Jacko Rhedoor Abodo

**Affiliations:** 1 Service de Médecine Interne et Gériatrie, Centre Hospitalier Universitaire de Treichville, Abidjan, Côte d'Ivoire,; 2 Service de Pédiatrie Hôpital Militaire d'Abidjan, Abidjan, Côte d'Ivoire,; 3 Service d'Endocrinologie, Diabétologie, Nutrition et Métabolisme, Centre Hospitalier Universitaire de Yopougon, Abidjan, Côte d'Ivoire,; 4 Service d'Endocrinologie, Centre Hospitalier Universitaire de Bouaké, Bouaké, Côte d'Ivoire

**Keywords:** Obésité, adolescent, scolaire, prévalence, Obesity, adolescent, school, prevalence

## Abstract

Peu d'études ont été consacrées à l'obésité du sujet jeune dans notre contexte malgré sa prévalence croissante. L'objectif de notre étude était donc de déterminer la prévalence et les facteurs associés à l'obésité de l'adolescent en milieu scolaire. Nous avons réalisé une étude multicentrique transversale au sein de quatre écoles dans la commune de Koumassi lors de la visite médicale des élèves. Ceux ayant des valeurs de l'indice de masse corporelle (IMC) supérieurs à 25kg/m^2^ ou ayant une courbe IMC/âge au-delà de IOTF 30 étaient retenus. La collecte des données s'est faite à l'aide d'un questionnaire et l'analyse statistique réalisé à l'aide du logiciel SPSS. Le test statistique utilisé était le test du Khi-deux (significativité P= 0,05). Au total 94 élèves avaient été recrutés soit une prévalence de 1,82% de surcharge pondérale. Il existait une prédominance féminine (73%) avec un sex ratio de 0,36 et un pic de prévalence entre 12 et 14 ans. Dans les antécédents, l'on notait que 16% des enfants avaient au moins un parent atteint d'obésité avec un niveau socio-économique bas dans 44% des cas. L'obésité était de grade I dans 45,7% des cas et le nombre moyen des repas était de deux repas quotidiens. Une activité sportive extrascolaire chez 41,5% des élèves. Les facteurs associés à la survenue de l'obésité étaient le sexe féminin (0,01), l'âge (0,02), le tour de taille (0,003) et le tour de hanche (0,005). L'obésité est une réalité en milieu scolaire. La lutte contre cette affection implique nécessairement une modification du style de vie combiné à l'action de divers acteurs de la santé publique.

## Abstract (en)

Few studies have focused on obesity in young people in our context, despite its increasing prevalence. The purpose of this study was to determine the prevalence of adolescent obesity in schools and identify associated factors. We conducted a multicenter cross-sectional study in four schools in the commune of Koumassi during students' medical check-ups. Adolescents with a body mass index (BMI) above 25 kg/m^2^ or a BMI-for-age curve beyond IOTF 30 were included. Data were collected using a structured questionnaire, and statistical analysis was performed with SPSS software. The chi-square test was used, with significance set at p= 0.05. A total of 94 pupils were recruited, representing a prevalence of overweight children of 1.82%. There was a female predominance (73%), with a sex ratio of 0.36, and a peak prevalence between 12 and 14 years. In family history, 16% of children had at least one parent with obesity, and 44% came from low socioeconomic households. Obesity was grade I in 45.7% of cases. The average number of daily meals was two, and 41.5% of students practiced extracurricular sports. Factors associated with the onset of obesity were female sex (p = 0.01), age (p = 0.02), waist circumference (p = 0.003), and hip circumference (p = 0.005). Obesity is a reality in school settings. Combating this condition necessarily involves lifestyle changes combined with action by various public health stakeholders.

## Introduction

L'obésité de l'enfant est un problème de santé grandissant. Les estimations font état de plus de 340 millions d'enfants et d'adolescents en surpoids ou obèses dans le monde, et si la tendance se poursuit, leur effectif atteindra 70 millions à l'horizon 2025 [1]. Longtemps relevée dans les pays industrialisés, la problématique liée à la surcharge pondérale est en constante ascension dans les pays en développement avec le phénomène de la mondialisation qui a induit la modification du style de vie. En Afrique subsaharienne, très peu de données sur l'obésité infantile sont disponibles, car les différentes actions relatives à la nutrition et à la santé publique avaient longtemps été axées sur la malnutrition et les problèmes de sécurité alimentaire [1]. Conscients du fait que la population en Côte d'Ivoire et même en Afrique est en majorité jeune, et connaissant les répercussions à long terme de l'obésité sur la santé, certains auteurs se sont intéressés à la question. En Côte d'Ivoire, l'une des premières études fut menée par Lokrou *et al*. [2] en 2008 qui avait relevé une prévalence du surpoids de 14,2% et celle de l'obésité 0,9% dans les établissements scolaires à Abidjan. Des travaux plus récents avaient été réalisés dans ce domaine [3,4] toutefois, ces données restent insuffisantes. Notre étude qui vient en complément des précédentes a pour objectifs de déterminer la prévalence et d'analyser les facteurs socio-démographiques et cliniques associés à la survenue de l'obésité de l'adolescent en milieu scolaire à Abidjan.

## Étude de cas

### Méthodes

**Type, période, contexte et population d'étude:** il s'agissait d'une étude transversale à visée descriptive et analytique réalisée dans la commune de Koumassi, l'une des treize communes du district d'Abidjan. Il s'agit d'un quartier mixte dont près d'un tiers des habitations sont de type précaire (26%) [5]. Notre étude avait été réalisée sur une période de deux mois (1^er^ mars au 29 avril 2022) au cours d'une visite médicale annuelle systématique des écoles. Durant cette période, quatre établissements avaient été visités selon le programme de passage établi. Il s'agissait d'un au sein de quatre établissements dont un établissement public et trois privés. Tous les élèves présents au cours de la visite médicale dont les valeurs de l'Indice de Masse Corporelle (IMC) étaient supérieures à 25kg/m^2^ pour les plus de 18 ans avaient été recrutés. Concernant les élèves de moins de 18 ans, ceux dont la courbe de l'IMC pour l'âge était située au-delà du 97^e^ percentile selon les normes définies pour l'âge et le sexe [6] avaient été recrutés.

**Déroulement de l'enquête:** la collecte des données était faite à l'aide de fiches d'enquête comportant: les paramètres sociodémographiques: âge, sexe, nationalité, lieu d'habitation, les antécédents et mode de vie (activité physique, travaux ménagers), niveau d'étude, la profession des parents, les habitudes alimentaires qui ont porté sur la fréquence de prise des principaux repas et l'existence de collations; les paramètres cliniques: poids, taille, calcul de l'IMC chez les plus de 18 ans, courbe IMC pour l'âge si l'âge était inférieur à 18 ans, tour de taille, tour de hanche, la pression artérielle, le poids et la taille des parents permettant le calcul de leur IMC Le poids (kg) avait été mesuré à l'aide d'une pèse personne manuel de marque SECA VIVA 750 et la taille à l'aide d'une toise. La pression artérielle avait été prise à l'aide d'un tensiomètre électronique à brassard (Spengler) avec coussinet gonflable; pour la classification de l'obésité et du surpoids, les références de *l'International Obesity Task Force* (IOTF) établies avaient été retenues [6]. Les seuils définissant le surpoids et l'obésité correspondaient aux centiles de l'IMC atteignant respectivement les valeurs 25kg/m^2^ (IOTFC-25) et 30kg/m^2^ (IOTFC-30) à 18 ans. Le surpoids était défini par des valeurs de l'IMC comprises entre 25 et 29,9kg/m^2^ chez les élèves de plus de 18 ans ou des valeurs de l'IMC situées entre les courbes de référence IOTF 25 et IOTF 30 chez les élèves de moins de 18 ans.

**Procédure d'échantillonnage**: il s'agissait d'un échantillonnage de convenance, constitué par la totalité des élèves présents le jour des différentes visites médicales effectuées au sein des quatre écoles durant la période définie.

**Définitions opérationnelles:** le terme adolescent dans notre étude comportait les enfants dont l'âge était compris entre dix et vingt-quatre ans. L'obésité grade I était définie par un IMC compris entre 30 et 34,9kg/m^2^ chez les élèves de plus de 18 ans ou des valeurs de l'IMC situées entre les courbes de référence IOTF 30 et IOTF 35 chez les sujets de moins de 18 ans. Obésité de grade II: L'IMC est situé entre 35 et 39,9kg/m^2^ chez les élèves de plus de 18 ans ou les valeurs de l'IMC sont comprises entre les courbes IOTF 35 et IOTF 40 chez les élèves de moins de 18 ans. Obésité de grade III: IMC supérieur à 40 kg/m^2^ chez les élèves de plus de 18 ans ou des valeurs de l'IMC situées au-delà de la courbe de référence IOTF 40.

**Saisie et analyse:** la présentation des résultats sous forme de tableaux ou graphiques a été effectuée avec le logiciel Excel. L'analyse des données avait été effectuée par le logiciel SPSS version 26 (IBM). La prévalence de l'obésité avait été calculée en effectuant le rapport du nombre d'élèves ayant des valeurs de l'IMC au-delà de 30kg/m^2^ (ou une courbe IOTF au-delà de 30) sur le nombre total d'élèves ayant pris part à la visite médicale, le tout multiplié par 100. Pour rechercher les facteurs influençant la survenue de l'obésité, l'échantillon avait été classé en deux catégories, le groupe des élèves en situation d'obésité avérée était comparé à ceux en surpoids. Différentes variables d'ordre sociodémographique, clinique avaient été comparées dans les deux groupes avec les tests statistiques. En analyse univariée, les variables quantitatives étudiées (âge, tour de taille, tour de hanche, le nombre de repas par jour) avaient été comparées par les tests d'ANOVA ou de Wilcoxon. Les variables qualitatives (catégorie socioprofessionnelle des parents, notion de surcharge pondérale chez les parents, activité extrascolaire, antécédents) avaient été comparées par le test du khi deux ou de Fisher en fonction de l'effectif. Le seuil de significativité était de p=0,05. Puis les variables significatives avaient fait l'objet d'une analyse multivariée afin de déterminer l'odds ratio.

### Résultats

#### Caractéristiques générales de la population

**Figure 1:**
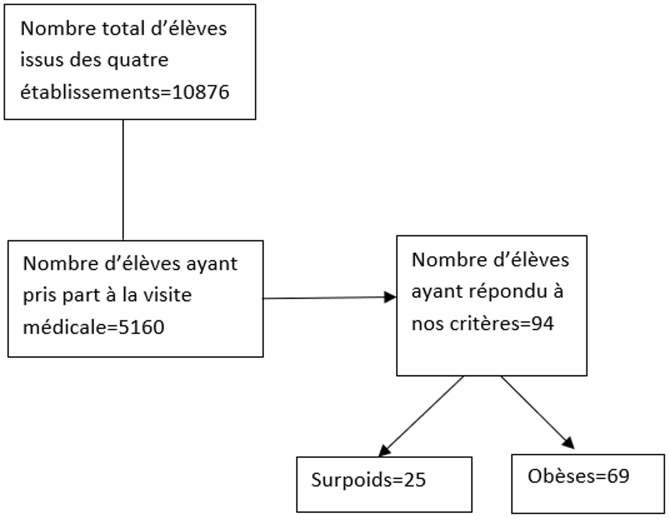
diagramme de flux des participants à l'étude, recrutés à partir de la visite médicale au sein de quatre établissements dans la commune de Koumassi de la période du 1^er^ mars 2022 au 29 avril 2022

Sur un total de 5160 élèves examinés durant la visite médicale, 94 présentaient un excès pondéral, soit une prévalence de 1,82% dont 1,33% d'élèves en situation d'obésité (Figure 1). L'âge moyen des élèves était de 14,5±2,4 ans avec une prévalence de l'obésité plus importante à l'âge de 12 et 14 ans (Tableau 1). On notait une prédominance féminine (73%) et les élèves résidaient pour la plupart (72%) dans la commune de Koumassi (Tableau 2). Près de la moitié (44,7%) des parents d'élèves en excès pondéral étaient des employés du secteur informel. L'on dénombrait 18% de cadres et 37,2% de fonctionnaires.

**Tableau 1:** prévalence du surpoids et de l'obésité selon de l'âge des élèves de notre échantillon recrutés au sein de la visite médicale annuelle au cours de la période du 1^er^ mars 2022 au 29 avril 2022

Age de la population	Effectif Total N = 94	Elèves en surpoids N (%)	Elèves en situation d'obésité N (%)
11 ans	11(11,70%)	4 (36,4%)	7(63,6%)
12 ans	15(15,9%)	6(40%)	9(60%)
13 ans	12(12,8%)	6(50%)	6(50%)
14 ans	17(18%)	5(29,4%)	12(70,6%)
15 ans	12(12,8%)	2(16,7%)	10(83,3%)
16 ans	8(8,51%)	0(0)	8(100%)
17 ans	7(7,44%)	0(0)	7(100%)
18 ans - 24 ans	12(12,8%)	2(16,7%)	10(83,33%)

**Tableau 2:** analyse des facteurs associés à la survenue de l'obésité chez les élèves de Koumassi recrutés au cours de la visite médicale annuelle sur une période de deux mois (1^er^ mars 2022 au 29 avril 2022)

Facteurs	Effectif ou moyenne	Elèves en surpoids N (%)	Elèves obèses N (%)	P
**Total**	94	25(26,59%)	69(73,04%)	-
**Sex**				
Féminin	69(73,4)	14(20,3)	55(79,7)	0,02
Masculin	25(26,6)	11(44)	14(56)	
**Age (années)**	14,54±2,4	13,28±2,09	15±2,39	0,01
**Etablissement**				
Privé	71(75,5)	19(26,8)	52(73,2)	0,94
Public	23(24,5)	6(26,1)	17(73,9)	
**Excès pondéral chez l'un des parents**				
Oui	17(18,1)	3(17,6)	14(82,4)	0,35
Non	77(81,9)	22(28,6)	55(71,4)	
**Catégorie socio-professionnelle**				
Cadre supérieur	17(18,1)	5(29,4)	12(70,6)	0,95
Fonctionnaire	35(37,2)	9(25,7)	26(74,3)	
Secteur informel	42(44,7)	11(26,2)	31(73,8)	
**Tour de taille (cm)**	99,7±15,6	89,44±13,24	103.46±14,8	0,003
**Tour de hanche (cm)**	108,05±12,8	97,92±9,77	111,72±11,72	0,005
**Nombre de repas par jour**	2,98±0,64	3,04±0,45	2,96±0,69	0,27
**Activité physique extrascolaire**				
Oui	39(41,5)	14(35,9)	25(64,1)	0,08
Non	55(58,5)	11(20)	44(80)	
**Heures passées devant la télévision par semaine**	8±5	7,81±5	9,68±6	0,18

Concernant le mode de vie, très peu d'élèves (2%) mangeaient à la cantine scolaire. Deux tiers des élèves (66%) prenaient deux repas quotidiens et 15% d'entre eux plus de trois repas quotidiens (Tableau 2). Plus des trois quarts des élèves (79,8%) pratiquaient le sport à l'école mais seulement 41,5% exerçaient en plus une activité sportive extrascolaire. Le football était le sport le plus pratiqué (32%) suivi des arts martiaux (20%), le basket ball et la gymnastique (10,26 % des cas chacun). S'agissant des comorbidités associées, l'asthme était la pathologie la plus fréquente (21,3%), suivi de l'ulcère gastroduodénal (4,25%), l'hypertension artérielle (1,1%), le diabète (1,1%). Un excès pondéral était noté chez respectivement 16% des pères et 11,7% des mères d'enfants obèses. Sur le plan clinique l'obésité de grade I était prédominante (45,7%) suivie du surpoids (26,6%) et de l'obésité de grade II (19,1%). On notait une augmentation du tour de taille (99,7±15,6 cm) et du tour de hanche (108,05±12,8 cm) (Tableau 2).

#### Facteurs associés au passage à l'obésité

Le Tableau 2 résume l'analyse des facteurs associés à la survenue d'une obésité. S'agissant des facteurs démographiques, le sexe féminin était associé à la survenue de l'obésité (P= 0,02). Un lien statistique était également retrouvé entre l'âge et la survenue d'une obésité (P= 0,01), les élèves en situation d'obésité étant en moyenne légèrement plus âgés (15ans±2,09 contre 13,28±2,09).

Cependant, la catégorie socioprofessionnelle des parents n'avait aucune influence sur la survenue de l'obésité (P= 0,95). Il en était de même pour le type d'établissement scolaire fréquenté, les élèves des écoles privées n'étant pas plus obèses que ceux du public. En ce qui concerne le mode de vie, les paramètres étudiés tels que le nombre d'heures passées devant la télévision (P= 0,18), l'existence d'une activité sportive extrascolaire (P= 0,08) et le nombre de repas quotidiens (P= 0,27) avaient peu d'influence sur la survenue de l'obésité. S'agissant des facteurs cliniques, seuls le tour de taille et le tour de hanche étaient statistiquement associés à la présence d'une obésité, respectivement P= 0,003 et 0,005. Aucun lien significatif n'avait été retrouvé entre la présence d'une surcharge pondérale chez les parents et la survenue de l'obésité chez les élèves de notre échantillon (P= 0,35). Après analyse multivariée les principaux facteurs associés à la survenue de l'obésité étaient le tour de taille (P= 0,025; OR= 1,18; intervalle de confiance= 1,008-1,12) et le tour de hanche (P= 0,001, OR= 1,11 intervalle de confiance: 1,04-1,19).

## Discussion

Cette étude avait pour objectifs principaux de déterminer la prévalence et les facteurs associés à la survenue de l'obésité de l'adolescent dans la commune de Koumassi. A la différence des pays industrialisés où l'obésité infantile constitue un problème de santé publique, l'obésité du sujet jeune dans notre contexte africain reste une affection très peu étudiée avec des données encore parcellaires, ce qui sous-estime la prévalence réelle de cette affection. La prévalence de l'obésité dans notre étude qui était de 1,33%, reflète le constat de la plupart des données africaines qui retrouvaient une faible prévalence de l'obésité [7]. Des proportions légèrement plus élevées avaient été relevées par Raiah *et al*. en Algérie [8] et Soumana *et al*. en 2021 [9] à Niamey: respectivement 5% et 6% d'obésité en milieu scolaire contre 4% et 9,5% d'élèves en surpoids. Cependant, leurs études intéressaient les élèves de l'école primaire à partir de l'âge de six ans; période propice au rebond d'adiposité pendant laquelle les enfants ont une légère tendance à la prise de poids par rapport à la taille [8].

Les facteurs associés à la survenue de l'obésité dans notre étude étaient le sexe féminin, l'âge, l'existence d'un tour de taille et d'un tour de hanche élevé. Après étude multivariée seuls les tours de taille et de hanche avaient été identifiés comme facteurs indépendants de la survenue de l'obésité. Sur le plan épidémiologique, l'obésité du sujet jeune a un profil quasi-identique à plusieurs données africaines [2,3,8,9] avec une nette prédominance féminine et un pic de prévalence au début de l'adolescence. La prévalence de l'obésité à cet âge peut être sans doute du fait de certains facteurs comportementaux: désordres alimentaires en partie dû au stress des examens de fin de cycle, mais aussi physiologique (stade pubertaire, imprégnation hormonale). L'obésité chez l'enfant présente en outre un risque important de persistance à l'âge adulte. De plus le contexte hormonal explique d'une part la prédominance féminine de l'obésité comme l'ont également notée plusieurs auteurs [8-10]; les femmes étant moins actives que les hommes dans notre contexte.

Bien que la notion d'obésité parentale ne soit pas significativement associée à la survenue d'une obésité chez les élèves, la surcharge pondérale notée chez 16% des parents d'élèves obèses pourrait témoigner de l'implication de facteurs génétiques dans la survenue de l'obésité. Une étude en Algérie avait conclu que les enfants avaient 2,2 fois plus de risque d'être en surpoids lorsqu'ils avaient un antécédent de surpoids chez l'un des parents [8]. A Abidjan, une étude plus récente avait retrouvé 61,9% d'excès pondéral chez les parents d'enfants en surpoids inscrits dans un programme de coaching en santé [4]. La relation intime entre l'adiposité parentale et celle des enfants s'explique par le partage des facteurs génétiques et des facteurs environnementaux dans la famille. Ce fait constitue un élément pronostique car le risque pour un enfant de devenir un adulte obèse augmente non seulement avec la sévérité de la définition de l'obésité, mais aussi avec l'obésité parentale.

En ce qui concerne le niveau socio-économique, dans notre étude, la catégorie socioprofessionnelle qui présage du niveau socioéconomique n'avait pas d'influence statistique sur la survenue de l'obésité. Cependant la plupart des données relèvent son impact sur la survenue de l'obésité ce qui explique d'une part la prédominance de la surcharge pondérale dans les établissements privés beaucoup plus coûteux que ceux du public [8,9,11]. Par exemple dans l'étude de Soumana *et al*. [9] au Niger ainsi que celle de Choukem *et al*. au Cameroun [12], un bon niveau socio-économique avait été identifié comme étant un facteur de risque d'obésité (OR= 4,61) contrairement aux Etats-Unis où les personnes ayant un niveau socio-économique bas étaient les plus concernées par l'obésité [13]. Il est clair que le développement socio-économique entraîne une occidentalisation du mode de vie avec la consommation régulière d'aliments industrialisés (fast-foods), une sédentarité accrue avec une augmentation de la fréquence des repas, certains élèves ayant droit en plus à des collations journalières en plus des trois principaux repas. Ceci a comme résultat que de nombreuses personnes ont un bilan énergétique positif avec une consommation de calories plus importante que celle dépensée.

Cette inégalité sur le plan socio-économique influe sans doute sur l'insuffisance de l'activité physique. En effet les personnes plus favorisées (niveau d'éducation, revenus et statut professionnel plus élevés) ont plus tendance à effectuer une activité physique régulière (jogging, natation, arts martiaux, volleyball) que ceux moins favorisés [14,15]. S'agissant du profil clinique, l'obésité était plus fréquente que le surpoids et était associée à une augmentation du tour de taille et du tour de hanche. Cette augmentation du tour de taille reflète le risque accru de complications cardiovasculaires. L'absence de stratification du risque cardiovasculaire de ces élèves constitue sans doute l'une des faiblesses de notre étude. La prévalence de l'hypertension artérielle était faible; cependant la recherche d'un prédiabète, ou même d'une dyslipidémie n'a pas été réalisée.

Divers auteurs se sont également intéressés à l'étude des facteurs prédictifs de la survenue de l'obésité. L'étude de Raiah *et al*. [8] en Algérie par exemple avait mentionné en plus du sexe féminin, un poids de naissance au-delà de 3,8kg, et les mauvaises habitudes alimentaires comme facteurs favorisants. Soumana *et al*. [9] rajoutaient la sédentarité et le niveau socio-économique des parents. Fofana *et al*. [11] soulignaient également l'impact psychologique sur la survenue de l'obésité. Ces constats sont en phase avec les données occidentales, ce pourquoi la Haute autorité de santé recommande une prise en charge holistique regroupant plusieurs domaines d'intervention avec pour objectif final la modification du mode de vie [6]. Parmi ces aspects suscités, la psychologie demeure la plus importante car les enfants et les adolescents qui sont en état d'obésité peuvent en subir des séquelles psychosociales nuisibles, ce qui peut impacter leur qualité de vie [16]. L'étude menée à Abidjan par Assi-Kaudjhis *et al*. confirme cette hypothèse [4] ; en effet, après des séances de coaching santé dans le cadre d'un programme pour adolescents, une amélioration de leur santé physique et psycho-sociale associée à une diminution de l'IMC allant de 31,3 à 27,8% avait été notée.

## Conclusion

L'obésité de l'adolescent est une réalité dans notre contexte. Notre étude réalisée au sein de différentes écoles de la commune de Koumassi est assez représentative de l'obésité de l'adolescent en milieu abidjanais caractérisé par sa prédominance féminine et son pic de fréquence en début d'adolescence. Ces données corroborent en effet celles de la littérature africaine. Cependant notre étude relève deux faits particuliers: la prédominance de l'asthme, peu décrite dans les données africaines et le bas niveau socio-économique retrouvé chez près de la moitié des parents d'enfants en excès pondéral contrairement aux données précédentes qui relevaient surtout le bon niveau socio-économique comme facteur d'obésité.
